# Smart intercropping system to detect leaf disease using hyperspectral imaging and hybrid deep learning for precision agriculture

**DOI:** 10.3389/fpls.2025.1662251

**Published:** 2025-10-07

**Authors:** S. B. Goyal, Varun Malik, Anand Singh Rajawat, Mudassir Khan, Amna Ikram, Bayan Alabdullah, Abrar Almjally

**Affiliations:** ^1^ Chitkara University Institute of Engineering and Technology, Chitkara University, Punjab, India; ^2^ School of Computer Science & Engineering, Sandip University, Nashik, Maharashtra, India; ^3^ Department of Computer Science, College of Computer Science, Applied College Tanumah, King Khalid University, Abha, Saudi Arabia; ^4^ Department of Computer Science and IT, Government Sadiq College Women University, Bahawalpur, Pakistan; ^5^ Department of Information Systems, College of Computer and Information Sciences, Princess NourahbintAbdulrahman University, Riyadh, Saudi Arabia; ^6^ College of Computer and Information Sciences, Imam Mohammad Ibn Saud Islamic University (IMSIU), Riyadh, Saudi Arabia

**Keywords:** intercropping system, maize–soybean, pea–cucumber, hyperspectral imaging, deep learning, precisionagriculture

## Abstract

**Introduction:**

The rapid growth of the global population and intensive agricultural activities has posed serious environmental challenges. In response, there is an increasing demand for sustainable agricultural solutions that ensure efficient resource utilization while maintaining ecological balance. Among these, intercropping has gained prominence as a viable method, promoting enhanced land use efficiency and fostering environment for crop development. However, disease management in intercropping systems remains complex due to the potential for cross-infection and overlapping disease symptoms among crops. Early and precise illness recognition is, therefore, critical for sustaining crop condition and efficiency.

**Methods:**

This study introduces an intelligent intercropping framework for early leaf disease detection, utilizing hyperspectral imaging and hybrid deep learning models for precision agriculture. Hyperspectral imaging captures intricate biochemical and structural variations in crops like maize, soybean, pea, and cucumber—subtle markers of disease that are otherwise imperceptible. These images enable accurate identification of diseases such as rust, leaf spot, and complex co-infections. To refine disease region segmentation and improve detection accuracy, the proposed model employs the synergistic swarm optimization (SSO) algorithm. A phase attention fusion network (PANet) is utilized for deep feature extraction, minimizing false detection rates. Furthermore, a dual-stage Kepler optimization (DSKO) algorithm addresses the challenge of high-dimensional data by choosing the most applicable landscapes. The disease classification is performed using a random deep convolutional neural network (R-DCNN).

**Results and discussion:**

Experimental evaluations were conducted using publicly available hyperspectral datasets for maize–soybean and pea–cucumber intercropping systems. The suggested ideal attained remarkable organization accuracies of 99.676% and 99.538% for the respective intercropping systems, demonstrating its potential as a robust, non-invasive tool for smart, sustainable agriculture.

## 1 Introduction

Agriculture is the primary source of food, revenue, and jobs and contributes significantly to the global economy. Agriculture generates 18% of the country’s GDP and raises the employment rate to 53% in India and other low- and middle-income nations with large numbers of farmers. Because crop diseases drastically lower production, they have become a nightmare (Jamjoom et al., 2023). When dealing with several illnesses or a variety of planting circumstances, the conventional approaches are not very flexible (Zhao et al., 2024). Improved decision-making in agricultural production management is facilitated by the early detection of plant diseases. In addition to a back propagation neural network, conventional approaches like SVM and K-means clustering algorithms (Li et al., 2022) have been employed for plant disease detection. Cucumber leaf disease is classified using a two-stage model that combines DeepLabV3+ and U-Net in complicated backdrops (Raza et al., 2025). The Dice coefficient for lesion segmentation was 0.6914, the accuracy for illness classification was 92.85%, and the accuracy for leaf segmentation was 93.27%. Expanders and feature extraction are performed on point cloud data using the Generate Adversarial-Driven Cross-Aware Network (GACNet) (Yang et al., 2024). Through the dynamic combination of geographical location and feature attributes, GACNet improves the effectiveness of feature extraction. A cascaded incremental region network (Inc-RPN) (Hai et al., 2025) is used for accurate apple leaf disease detection in natural settings. A Coffee-Net model is used for accurate classification of coffee leaf diseases. Coffee-Net achieves 99.95% accuracy, outperforming ANN, Mask R-CNN, MobileNetV2, and ResNet50 by 0.6%, 4.32%, 0.02%, and 1.95% respectively (Zhang et al., 2023). The InceptionV3, MobileNetV1/V2, and VGG-16 models are optimized using pruning and quantization-aware training (Zhang et al., 2024). Although existing models demonstrate high accuracy in plant disease detection, they depend on large, labeled datasets and lack adaptability to new disease classes with limited data. To address these limits, this study presents a smart intercropping system by hyperspectral imaging and hybrid deep learning for accurate leaf disease detection and enhanced precision agriculture (Bidarakundi and Kumar, 2024; Da Silva and Almeida, 2024). Hyperspectral imaging captures intricate biochemical and structural variations in crops like maize-soybean and pea-cucumber, subtle markers of disease that are imperceptible. The key contributions of the planned work are given as trails.

1. The synergistic swarm optimization (SSO) algorithm is used to accurately segment the diseased regions from hyperspectral images of leaves. By focusing on the most relevant regions of infection, SSO enhances the spatial precision of the extracted disease areas, which improves the downstream classification accuracy.
2. To extract deep features from the segmented disease regions, phase attention fusion network (PANet) is integrated into the framework.
3. To tackle high-dimensionality issues inherent in hyperspectral data, the dual-stage Kepler optimization (DSKO) algorithm is used for feature optimization.
4. The random deep convolutional neural network (R-DCNN) model is used to classify leaf diseases within intercropping system, includes maize–soybean and pea–cucumber combinations which contributes to disease prediction even under complex conditions with co-occurring infections.
5. Experimental samples were collected on September 13, 2023, from maize–soybean and pea–cucumber intercropping fields in Fei Cheng City, Tai’an City, Shandong Province, China (Liu et al., 2024), ensuring the practical applicability of the framework.

The rest of this paper is organized as follows. Section 2 discusses the recent works on leaf disease prediction. The proposed methodology for smart intercropping system for accurate leaf disease detection is presented in Section 3. The consequences and conversation are presented in Section 4. The paper concludes in Section 5.

## 2 Related work

TinyResViT (Truong-Dang et al., 2025) is a lightweight efficient hybrid model that combines residual net (ResNet) and vision transformer (ViT) for leaf disease detection. The superfluous model weights are removed using the downsampling block that connects ViT and ResNet. With F1-scores of 97.92% and 99.11%, respectively, TinyResViT performs better on the plant village and Bangladeshi agricultural disease datasets. MobileH-Transformer (Thai and Le, 2025) combines a convolutional neural network (CNN) and Transformer for accurate leaf disease detection with minimal computation demands. The model obtains competitive F1-score values of 97.2% on the Maize leaf disease dataset and 96.80% on the Plant Village dataset, according to the results on publicly available datasets. A lightweight grape disease recognition method based on the GC-MobileNet model, for classification and fine-grained grading of diseases (Canghai et al., 2025). With an accuracy of 98.63 percent, GC-MobileNet outperforms MobileNetV3 by 6.51%. In grape vineyards, a real-time leaf detection system is used to identify and spray ill leaves, improving the efficacy of pesticide treatment (Khan et al., 2025). A deep learning algorithm can identify and classify six distinct diseases that affect potato leaves: nematodes, bacteria, viruses, fungi, phytophthora, and pests (Mhala et al., 2025). The LBPAttNet model integrates a lightweight coordinate attention mechanism into ResNet18 to enhance disease localization and reduce background interference (Wu P. et al., 2025).The model outperforms ResNet18 by 3.84% and 2.59%, respectively; with accuracy rates of 92.78% and 98.13%. Areas of interest in the crop leaf photos are found using an enhanced version of the U-Net segmentation algorithm (Chavan et al., 2025).The model’s F-measure was around 0.956 and its detection accuracy was better at 0. 982. For the accurate identification of grapevine leaf and fruit diseases, the ResNet50 model was improved using batch normalization (Sagar et al., 2025). During the validation stages, the model’s accuracy in distinguishing between healthy and sick grapevine leaves was 95%. LGENetB4CA combines modified EfficientNetB4 model with the LeafGabor filter (Van et al., 2025) which employs coordinate attention block to efficiently collect both spatial and channel-wise information.LGENetB4CA obtained an accuracy of 85.90% on COLD chili and 89.61% on JNUCLS for the COLD chili dataset. A semi-supervised method using modified pyramid scene parsing network (PSPNet) (Fan et al., 2025) for segmenting apple leaves. A fine-grained multi-label model based on transformers is used to categorize illnesses of apple leaves.

### 2.1 Problem description

**Figure 1:**
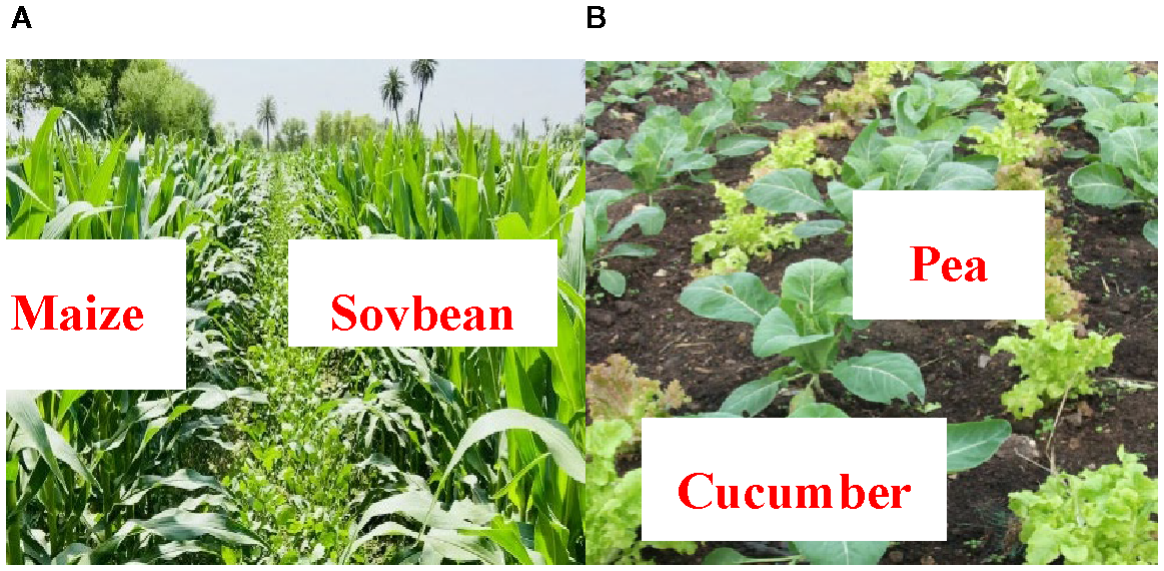
Interconnect fields of **(A)** Maize-soybean and **(B)** Pea-cucumber.

Form related works, and numerous deep learning models (Liu et al., 2024; Dhanka and Maini, 2025; Han et al., 2025; Kamonsukyunyong et al., 2025; Logeswari et al., 2025; Mu et al., 2025; Patel, 2025; Qiao et al., 2025; Zhang C. et al., 2025; Zhang Z. et al., 2025) that have demonstrated promising results in the field of leaf ailment finding have been developed under monoculture conditions and exhibit limitations (**Table 1**) when applied to diversified cropping systems (Qin et al., 2024; Xu et al., 2024; Liang et al., 2025; Mohanty et al., 2025; Xu et al., 2025). TinyResViT, MobileH-Transformer, and GC-MobileNet have shown high accuracy on benchmark datasets; however, these methods rely heavily on ideal conditions and not generalize to real-world intercropping systems (Upadhyay et al., 2025). A critical observation from the literature review indicates that most existing works do not address leaf disease prediction in intercropping systems. To date, few studies have attempted to explore disease detection (Liu et al., 2024) within such systems, despite their increasing relevance in sustainable agriculture (Xu et al., 2023; Gong et al., 2024; Tu et al., 2024; Wang et al., 2024; Lu et al., 2025). Intercropping systems introduce unique challenges such as overlapping foliage, interspecies spectral interference, and a higher risk of co-infections—factors that are typically overlooked in conventional monoculture-based models (Liu et al., 2024; Qin et al., 2024; Xu et al., 2024; Cai et al., 2025; Chi et al., 2025; Christy and Jeyaraj, 2025; Dhanka and Maini, 2025; Han et al., 2025; Kamonsukyunyong et al., 2025; Liang et al., 2025; Li J. et al., 2025; Lingayya et al., 2025; Li Q. et al., 2025; Logeswari et al., 2025; Maranga et al., 2025; Mohanty et al., 2025; Mu et al., 2025; Patel, 2025; Qiao et al., 2025; Ratmele et al., 2025; Wang and Ruan, 2025; Wu M. et al., 2025; Xu et al., 2025; Zhang C. et al., 2025; Zhang Z. et al., 2025). To fill the research gaps, an intelligent framework for integrated early detection of foliar diseases is proposed, combining hyperspectral imaging with hybrid deep learning applied to precision agriculture. Hyperspectral imaging provides rich spectral information, capable of capturing subtle biochemical and structural changes in crops such as corn-soybean and pea-cucumber (**Figure 1**).

**Table 1:** Summary of research gaps from existing state-of-art works on leaf disease detection and classification.

Ref.	Crop type	Disease class	Technique	Dataset used	Findings	Research gaps
(Truong-Dang et al., 2025)	Corn leaf	Healthy and non-healthy	ResNet, ViT	PlantVillage and Bangladeshi crops	F-measure 97.92% and 99.11%	Suffering from poor generalization and low efficiency
(Thai and Le, 2025)	Corn leaf	Healthy and non-healthy	MobileH-Transformer	PlantVillage	F-measure 97.2%	Less reliable when representing local spatial attributes
(Canghai et al., 2025)	Grape leaf	Grey spot, mosaic, and rust	GC-MobileNet, LeakyReLU	Grape leaf disease dataset	Accuracy 98.63 %	Lighting variations reduce classification accuracy
(Khan et al., 2025)	Grape vineyard leaf	Grey spot, mosaic, and rust	YOLOv7	Vineyard and Labeling	Mean average precision 64.6%	Require a large amount of data for training
(Mhala et al., 2025)	Potato leaf	Healthy and non-healthy	DenseNet201, ResNet152V2, and NasNetMobile	Synthetic dataset of 3076 images	Accuracy of 81.31%	Model scalability environments remain largely unexplored
(Wu P. et al., 2025)	Tea leaf	Healthy and non-healthy	LBPAttNet and ResNet18	Synthetic tea leaf dataset	Accuracy 92.78% and 98.13%	Overfitting when maximum deeper networks
(Chavan et al., 2025)	Crop leaf	Grey spot, mosaic, and rust	Improved U-Net and Local Gabor XOR	PlantVillage	Accuracy 98.2%, F-measure 95.6%	Class imbalance severely impacts the performance
(Sagar et al., 2025)	Grapevine leaf	Healthy, Downy, Powdery mildew	ResNet50	Synthetic dataset of 1,226 images	Precision94%, recall 96%	Lack of universality and poor migration capabilities
(Van et al., 2025)	Leaf of chili germplasm	Healthy and non-healthy	EfficientNetB4 with Coordinate Attention	JNUCLS and COLD chili	Accuracy of 89.61% and 85.9%	Not considering the overfitting and reducing time complexity
(Fan et al., 2025)	Apple leaf	Blotch, brown spot, grey spot, mosaic, and rust	Modified PSPNet	PlantVillage	Accuracy 96.35% and F-measure 91.28%	Suffer from subjectivity, inconsistency, and time consumption

## 3 Materials and methods

**Figure 2:**
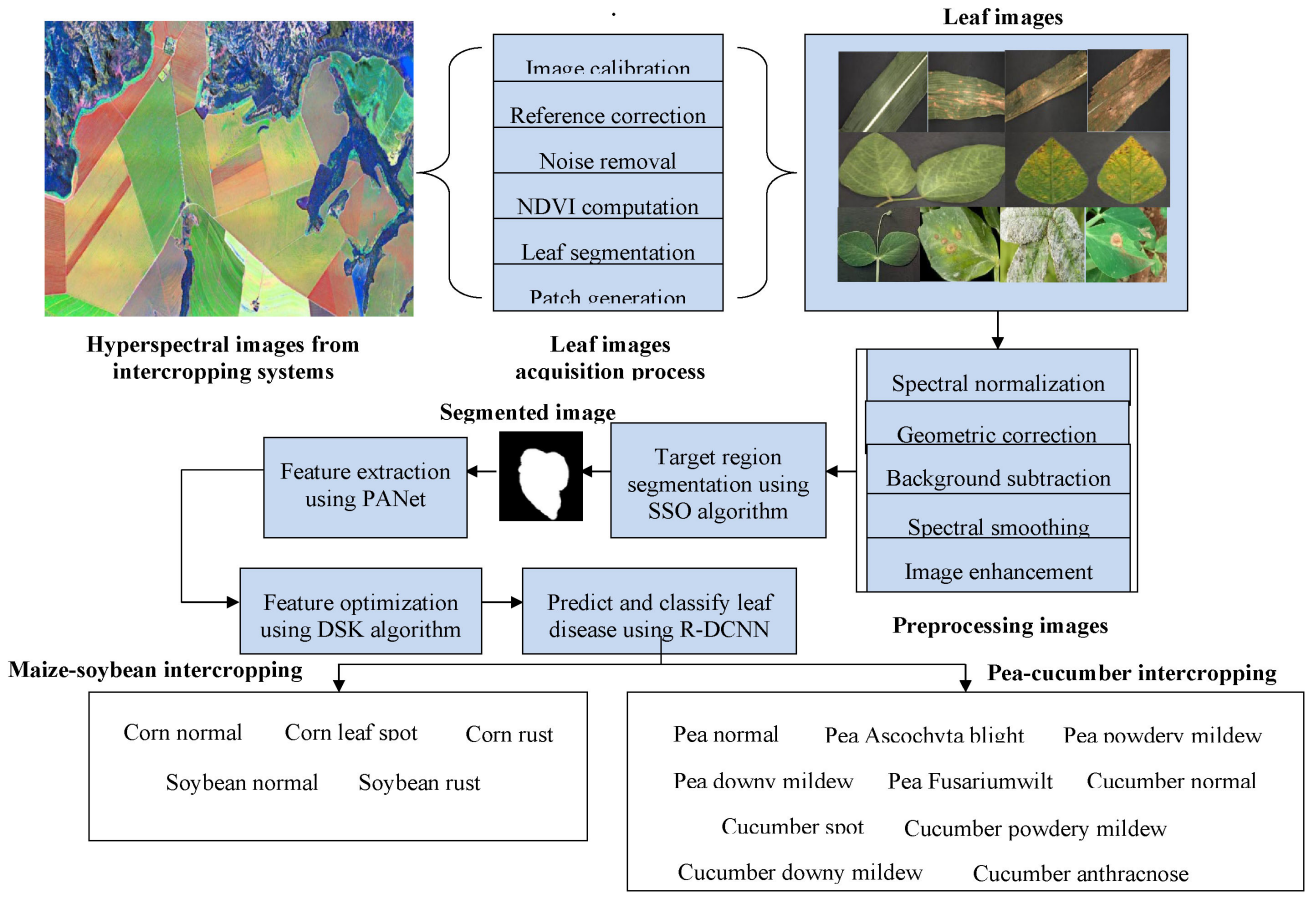
Smart intercropping system for leaf disease prediction.

This study involves collecting hyperspectral images from Maize–soybean and pea–cucumber intercropping fields. Leaf samples, including both healthy and diseased ones, were captured under controlled lighting conditions using a hyperspectral imaging setup. The raw hyperspectral data underwent a series of preprocessing steps such as image calibration, noise removal, normalization, and enhancement to improve image quality. Leaf regions were segmented, and relevant features were extracted and refined. These processed features were then castoff to classify and classify various leaf ailments present in the intercropping systems. The overall architecture of the proposed smart intercropping system for leaf disease prediction is presented in **Figure 2**. Hyperspectral and leaf images are acquired from maize–soybean and pea–cucumber intercropping systems, where calibration, reference correction, noise removal, NDVI computation, leaf segmentation, and patch generation are applied to prepare the data. The acquired images undergo preprocessing steps such as spectral normalization, geometric correction, background subtraction, spectral smoothing, and image enhancement, ensuring uniform quality and reducing distortions. After preprocessing, the SSO algorithm segments the diseased regions from the healthy tissues, producing precise masks that highlight only the infected areas. These segmented regions are then processed through the phase attention fusion network (PANet) to extract discriminative deep features, while the dual-stage Kepler optimization (DSKO) algorithm reduces redundancy and optimizes the feature space. The refined features are classified using the random deep convolutional neural network (R-DCNN), which predicts crop-specific diseases. Within the maize–soybean intercropping system, the categories include normal, leaf spot, and rust, whereas in the pea–cucumber system, multiple diseases such as Ascochyta blight, powdery mildew, downy mildew, Fusarium wilt, cucumber spot, and anthracnose are identified. This integrated pipeline establishes a coherent flow from image acquisition and preprocessing to segmentation, feature extraction, optimization, and classification, thereby enabling accurate and scalable disease detection in diverse intercropping environments.

### 3.1 Data collection and preprocessing

**Figure 3:**
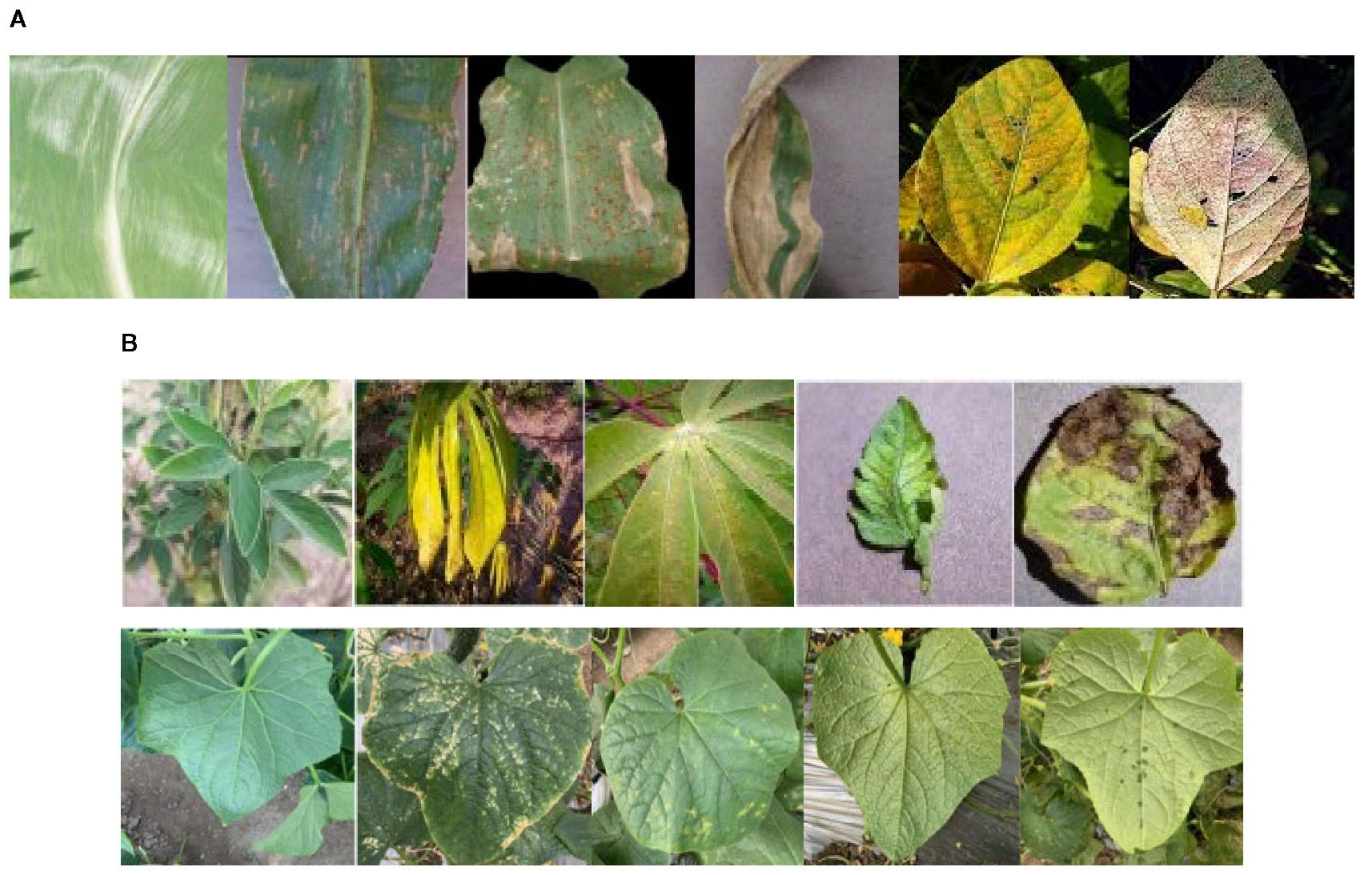
Sample leaf images from **(A)** Maize–soybean and **(B)** pea–cucumber intercropping fields with the disease class of corn normal, corn leaf spot, corn rust, corn hybrid, soybean normal, soybean rust” in Maize–soybean intercropping field; disease class of “pea normal, pea Ascochyta blight, pea powdery mildew, pea downy mildew, pea Fusarium wilt, cucumber normal, cucumber angular leaf spot, cucumber powdery mildew, cucumber downy mildew, and cucumber anthracnose” in pea–cucumber intercropping field.

The dataset used in this study is the publicly available hyperspectral dataset curated by Liu et al (Liu et al., 2024; Han et al., 2025; Zhang Z. et al., 2025). This dataset was collected from maize–soybean and pea–cucumber intercropping fields in Fei Cheng City, Tai’an, Shandong Province, China. It includes hyperspectral images of maize, soybean, pea, and cucumber leaves under various disease conditions. Specifically, the maize–soybean dataset contained healthy maize leaves, maize leaves with leaf spot, rust-infected maize leaves, and samples with combined infections, along with healthy and rust-infected soybean leaves. The pea–cucumber dataset (**Figure 3**) comprised healthy pea leaves as well as leaves infected with Ascochyta blight, powdery mildew, downy mildew, and Fusarium wilt. Cucumber samples included healthy leaves and those affected by angular leaf spot, powdery mildew, downy mildew, and anthracnose.

**Figure 4:**
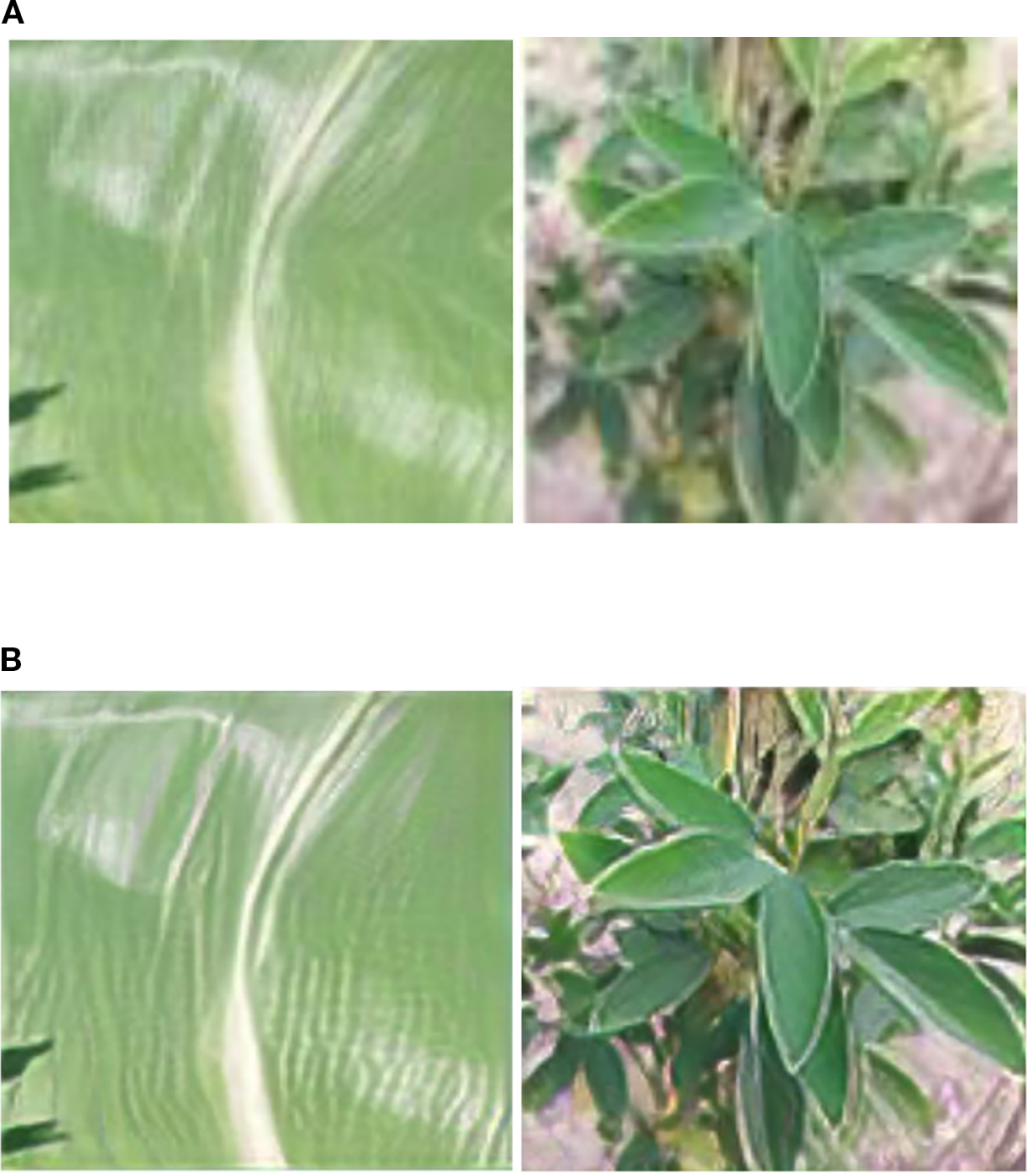
Example leaf images from the dataset: **(A)** raw input images of healthy and diseased leaves from maize–soybean and pea–cucumber intercropping systems; **(B)** corresponding preprocessed images after patch generation, normalization, and enhancement, illustrating the preparation of data for hyperspectral model training.

- The ground-truth labeling of these datasets was performed by experienced plant pathologists as reported by Liu et al (Liu et al., 2024; Han et al., 2025; Zhang Z. et al., 2025), who applied phenotypic criteria such as lesion shape, size, color, and distribution on leaves for accurate annotation. In this study, we directly utilized these expert-verified labels for model training and evaluation.
- Details of hyperspectral imaging instrumentation and acquisition protocols are available in Liu et al (Liu et al., 2024; Han et al., 2025; Zhang Z. et al., 2025). In this work, we focused on preprocessing, model training, and evaluation using the curated dataset. To address potential class imbalance, patch generation was applied during preprocessing, ensuring sufficient representative samples for each disease category. The final dataset was split using stratified 10-fold cross-validation, which preserved the proportion of classes in training and testing sets while reducing bias in performance evaluation.
- During model training, hyperparameters such as learning rate, batch size, and number of epochs were tuned experimentally. A grid search strategy was employed, with the learning rate selected in the range 10^-5^ to 10^-^³, batch size varied between 16 and 64, and the epoch count adjusted to ensure convergence without overfitting. These design choices were based on preliminary trials and prior studies in hyperspectral plant disease detection.
- To illustrate the preprocessing workflow, **Figure 4** presents representative raw images from the Liu et al. dataset alongside preprocessed images generated in this study, including patch extraction, normalization, and enhancement. **Table 2** summarizes the key spectral bands identified in the dataset, highlighting differences between healthy and diseased leaves in the visible (450–700 nm) and near-infrared (700–740 nm) ranges, which correspond to physiological changes such as chlorophyll reduction, red edge shifts, and water stress. **Table 3** reports the distribution of hyperspectral images across different crop and disease categories, with stratified splits used to ensure balanced representation in training, validation, and testing.

**Table 2:** Significant hyperspectral wavelengths distinguishing healthy and diseased leaves across maize–soybean and pea–cucumber intercropping systems, highlighting spectral variations associated with different plant diseases.

Crop	Disease	Wavelengths (nm)	Spectral feature	Comments
Maize	Healthy	450–500, 680–700	High chlorophyll reflectance	Reference baseline
	Leaf Spot	540–580, 700–740	Decreased chlorophyll, increased water stress	Clear difference from healthy
	Rust	550–600, 720–740	Red edge shift, higher reflectance in NIR	Indicative of pathogen infection
	Combined Infection	540–580, 680–740	Mixed spectral signatures	Strong variation across NIR
Soybean	Healthy	450–500, 680–700	High chlorophyll reflectance	Reference baseline
	Rust	550–600, 710–730	Red edge shift	Disease signature observable
Pea	Healthy	450–500, 680–700	High chlorophyll reflectance	Reference baseline
	Ascochyta Blight	530–570, 700–730	Reduced chlorophyll, slight NIR increase	Distinct from healthy
	Powdery Mildew	550–600, 710–740	Increased reflectance in NIR	Disease effect on leaf surface
Cucumber	Healthy	450–500, 680–700	High chlorophyll reflectance	Reference baseline
	Angular Leaf Spot	540–580, 710–740	Red edge shift	Clear spectral change
	Anthracnose	550–600, 720–740	Reduced chlorophyll, NIR variation	Distinct spectral pattern

**Table 3:** Dataset distribution for maize–soybean and pea–cucumber intercropping systems.

Crop	Disease	Training images	Validation images	Testing images	Total images
Maize	Healthy	500	100	100	700
	Leaf Spot	450	90	90	630
	Rust	460	92	92	644
	Combined Infection	400	80	80	560
Soybean	Healthy	480	96	96	672
	Rust	420	84	84	588
Pea	Healthy	500	100	100	700
	Ascochyta Blight	450	90	90	630
	Powdery Mildew	440	88	88	616
	Downy Mildew	430	86	86	602
	Fusarium Wilt	420	84	84	588
Cucumber	Healthy	500	100	100	700
	Angular Leaf Spot	450	90	90	630
	Powdery Mildew	440	88	88	616
	Downy Mildew	430	86	86	602
	Anthracnose	420	84	84	588

### 3.2 Target disease region segmentation

The target disease region segmentation plays a vital role by isolating only the infected portions of crop leaves from hyperspectral images while discarding irrelevant background and healthy regions. This step confirms that the subsequent stages of deep feature extraction and classification focus solely on the areas exhibiting disease symptoms, thereby improving accuracy and reducing false detections. To achieve this, a hybrid Synergistic Swarm Optimization (SSO) based on the Crowd Synchronization Algorithm (CSA) is employed (Logeswari et al., 2025). It is inspired by the collective behaviors of birds, fish, and ants, which rely on synchronization and cooperation to achieve optimal solutions. In this context, the input consists of hyperspectral images of maize, soybean, pea, and cucumber leaves, where each pixel carries intensity values across multiple spectral bands in the 400–1000 nm range. Unlike conventional segmentation methods such as K-means or Otsu’s thresholding, which fail under complex intercropping conditions, or deep segmentation models like U-Net that demand extensive annotated datasets and heavy computation, the proposed SSO-CSA hybrid dynamically adapts segmentation boundaries based on spectral similarities, swarm synchronization, and information sharing among candidate solutions (Zhang C. et al., 2025). This prevents premature convergence and ensures robust detection even in the presence of overlapping infections and multi-crop variations. The output of segmentation (Equation 1) process is a binary disease mask that highlights infected regions while suppressing background and healthy tissues, along with segmented hyperspectral sub-images that are passed to the PANet module for deep feature extraction. By focusing only on disease-affected regions, this segmentation approach reduces computational overhead and enhances the downstream classification accuracy of the system.


(1) P=Rand(B, dim)∗(un−ln)+ln

A matrix *P* represents fitness solution form the objective function (Equation 2).


(2) $$P=\left[\begin{array}{l} p_{1,1}\;\cdots \;p_{1,\;\dim}\\ \vdots \;\ddots \;\vdots \\ p_{B,1}\;\cdots \;p_{B,\;\dim} \end{array}\right]$$

where, un and ln stand for the vectors above and below each dimension of the issue space, respectively, and B implicitly represents the dimensions or variables Equation 3 of the given problem. Candidate solutions are updated with (*P*) (Dhanka and Maini, 2025).


(3) PNew(h, g)=P(h,g)+V(h,g)

where PNew(h, g) denotes the original optimal place g of the h-th applicant explanation, P(h,g) denotes g the current position of the h-th contender key, V(h,g) and g denotes the place of the h-th contender resolution value. The particles are based on the local and global attraction of states (Equation 4) (Kamonsukyunyong et al., 2025).


(4) $$V_{new}\left(h,g\right)=iwv+pbc+gbc+dac+anic+mdc$$

The $V_{new}\left(h,g\right)$ value is the disinterest weight value (IWV), describes as trails (Equation 5):


(5) iwv=z(s)∗V(h,g)

When the inertia weight parameter (z), which dynamically regulates the ratio of exploration to exploitation (Equation 6), has an adaptive mechanism denoted by *z*. The following formula is used to get the personal best coefficient (PBC) (Patel, 2025):


(6) $$pbc=R1*\left(Eps*Rand\left(xbest\right)-P_{h}\right)$$

where, $P_{h}$ solution number *h* is returned, *Rand(xbest)* is a unplanned resolution from the available entrant solutions, *R1* is a random value, and Eps returns a tiny assessment. The global best coefficient (*gbc*) Equation 7 is calculated as follows (Qiao et al., 2025):


(7) $$gbc=R2*jbest_{s}-P_{h}$$

where, *R2* is an accidental value $jbest_{s}$ representing the best comprehensive explanation and $P_{h}$ gives the solution number. The diversity preservation constant (*dmc*) is designed as trails (Equation 8) (Mu et al., 2025):


(8) $$dmc=R5*\frac{Diversity_{h}}{d2}-P_{h}$$

where, *d2* is an extra hastening factor for the variety period and *R5* is a random value. $Diversity_{h}$ stands for the location in the cluster where the particle’s neighborhood diversity is maximized. The research gaps discussed in **Table 1**. **Algorithm 1** describes the working process of target region segmentation using SSO.

Algorithm 1 **Target region segmentation using SSO.**


Input: Leaf image region, swarm parameters, fitness function criteria
Output: Target region segmentation
1. Begin;
2. Create random locations for every swarm particle.
3. The optimization process begins with a random guess of candidate solution
4. For s = 1 to S do
5. Compute the local and global attraction of states
6 The inertia weights can be rationalized at every iteration by the adaptive solution.
7. Update velocity of SSO
8. Find final display global best position
9. Find final display global best fitness
10. End if
11. Find the best putout value

### 3.3 Feature extraction

The feature extraction from the segmented target region of the disease transforms it into a set of meaningful descriptors that describe essential characteristics such as shape, texture, color, and structural patterns of the damaged area. In order for classification algorithms to accurately identify the type and severity of the disease, these extracted features are essential inputs. The fuzzy attention network (PANet) is used to efficiently extract features from the segmented disease areas in leaf images. PANet permits the network to focus more precisely on the most relevant input areas, such as diseased parts, while reducing the effect of healthy or non-relevant areas. Fuzzy attention components (Wang and Ruan, 2025) allow the model to selectively focus on exact feature maps that capture various visual cues such as texture, edges, and gradients at different levels of abstraction.

### 3.4 Feature optimization

In the context of identifying plant diseases, feature extraction often leads to a database that contains a variety of in-depth features. However, not all of these features are relevant or necessary for the classification of diseases. Feature optimization aims to reduce the dimensions of the dataset by identifying and retaining only the most important features that contribute efficiently to the classification task. By removing the less useful features, the model becomes more efficient, faster, and less prone to overfitting, thereby improving its performance and generalization capability. To optimize the features in this work, the two-stage Kepler optimization algorithm (DSKO) is used. DSKO is a metaheuristic algorithm inspired by nature and based on Kepler’s laws of planetary motion, which determine the movement of celestial bodies in space (Cai et al., 2025). The populace size is the numeral of planets $B_{x}$ that reflect the optimization problem’s decision parameters, is dispersed randomly over fuzzy sizes in the manner described in Equation 9:


(9) $${\vec{P}}_{h,g}(0)=R_{1}\times {\vec{P}}_{g,UP}+{\vec{P}}_{g,\;LOW}(1-R_{1}),h=1:B_{x};g=1:\dim$$

where h-th represents $P_{h,g}$ candidate solution, $B_{x}$ is the numeral of applicant solution in the exploration space *R_1_;* $P_{g,\;LOW}$ and $P_{g,UP}$ denotes the lesser and higher bounds of the g-th optimal parameter, separately. Where ai is the elliptical orbit semi-major axis at time s of object h, which is determined by Kepler’s third law as follows: the minimum value to prevent a divide-by-zero error. An absolute value randomly generated using a regular circulation to signify the orbital period of the objective. $P_{t}$ and $P_{h}$ ; denotes Euclidean distance normalization (Equation 10); defined as follows.


(10) $$r_{h-norm}(s)=(r_{h}(s)-r_{Min}(s))/(r_{Max}(s)-r_{Min}(s))$$

To inform the distance position of every objective from the Sun according to Equation 11 the previous steps:


(11) $$\begin{array}{c} {\vec{P}}_{h}(s+1)={\vec{P}}_{h}(s)+{\vec{v}}_{h}(s)\times f+({\vec{P}}_{h}(s)-{\vec{P}}_{h}(s))\times \\ \vec{u}\times (fj_{h}(s)+|R|) \end{array}$$

where ${\vec{P}}_{h}(s+1)$ denotes an object H’s new position at time s + 1, $P_{h}(s)$ denotes the object h’s current location at time *s*, VHS shows the velocity required for object h to move to the innovative situation, $P_{h}(s)$ displays the optimal sun place, which is linked to the greatest explanation with the lowest fitness score, and *F* is shown as a flag to change the route of the exploration.The normalized values of $Ab_{t}$ and $ab_{h}$ respectively (Equation 12),


(12) $$Ab_{t}=R_{2}\times (Fit_{t}(s)-Worst(s))/\sum_{K=1}^{B_{x}}\left(Fit_{K}(ab_{h})-Worst(s)\right)$$

where $Fit_{K}(ab_{h})$ is the value of the fitness function with respect to each position of the object K at the current time s; Worst(t) denotes the solution candidate with the greatest fitness score (Equation 13). The term ( $rb_{h}$ ), which denotes the normalized value of ( $r_{h}$ ), may be used to determine the Euclidian distance between $P_{h}$ and $P_{t}$ .


(13) $$rb_{h}(s)=||P_{t}(s)+P_{h}(s)|{|}_{2}=\sqrt{\left(\sum_{g=1}^{\dim}{\left(P_{T}(s)+P_{h}(s)\right)}^{2}\right)}$$

To accomplish exploration correctness, *µ(s)* is a purpose that exponentially debilities with time (*s*). The fitness optimization is expressed in Equation 14:


(14) $$\begin{array}{c} {\vec{P}}_{h,new}(s+1)\\ =\left\{ \begin{array}{l} {\vec{P}}_{h}(s+1)iffit\left({\vec{P}}_{h}(s)\right)\geq fit\left({\vec{P}}_{h}(s+1)\right)\\ {\vec{P}}_{h}(s) \end{array}\right. \end{array}$$

The procedure of feature optimization with DSKO is explicated in **Algorithm 2**.

Algorithm 2 **Feature optimization using DSKO.**


Input: Number of features, initial population matrix, maximum number of iterations
Output: Best optimal features
1. Initialize the population matrix with probable solutions
2. The randomly distributed over fuzzy rule-set
3. While do
4. The elliptical orbit semi major axis at time s of article h, by Kepler third law
5. The universal law of gravity is used to compute this force.
6. A cyclic regulatory parameter is compute by using the threshold set
7. Update the fitness value
8. An elite system is used to ensure optimal alignment of the Sun and planets.
7. End if
8. Find the best output value
9. End

### 3.5 Leaf disease prediction

Leaf disease prediction is process in the early detection and organization of plant diseases, based on the visual symptoms observed on the leaves of plants. In such an intercropping environment, the prediction of foliar diseases becomes more difficult due to the presence of various plants with different disease susceptibility profiles. Therefore, accurate disease prediction is crucial to prevent significant yield losses and ensure prompt intervention through appropriate disease management strategies. To achieve accurate and efficient predictions of leaf diseases in intercropping systems, a randomly used deep convolutional neural network (R-DCNN) is employed. R-DCNN is an advanced variant of the traditional deep convolutional neural network (DCNN), designed to enhance model performance by introducing random elements into the network structure and learning pathways. In R-DCNN, the convolution layer is used to learn parameters such as the weight matrix (n) and the dependence terms (*E*) of the convolution kernel (*k*). The complete convolution calculation $\omega_{x}$ is discussed in Equation 15 as follows (Li J. et al., 2025).


(15) $$\omega_{x}=E\sum_{L=0}^{l-1}\sum_{K=0}^{k-1}w_{x+L+y+K}n_{LK}+d$$

The following is the sample size of 1 × 2 if the pooling layer (*d*) employs uniform sampling $\omega_{xy}$ (Equation 16).


(16) $$\omega_{xy}=\frac{1}{s_{1}s_{2}}\sum_{L=y}^{s_{1}-1}\sum_{K-0}^{s_{2}-1}w_{X^{*}s_{1+y^{*}s_{2+K^{*}}}}$$

For the pooling layer, *s_1_* and *s_2_* stands for the random input and output values, respectively. A feature network of R-DCNN is mapping of features from an input image to convolutional kernel and a processing function in convolutional layer (Equation 17).


(17) $$w_{L}^{h}=E\left(\sum_{L=1}^{y_{K}^{L-1}}n_{L,k}⊗w_{K}^{h-1}+d_{K}^{h}\right)$$

To determine the neural output by using the nonlinear activation function is $d_{K}^{h}$ : The current layer additive bias k-th feature map is signified by E is the stimulation function $d_{K}^{h}$ (Equation 18) which usually starts at 0 (Chi et al., 2025).


(18) E(w)=Max(0,w)

The analysis’s findings demonstrate that the ReLU feature enhances the network’s capacity for recognition and learning. A fully connection layer $\varphi_{K}^{h}$ is used to finalizes the random of nonlinear mapping and network size optimization (Equation 19).


(19) $$\varphi_{K}^{h}=E\left(\sum_{l=1}^{y}w_{L}^{\left(h-1\right)}\cdot n_{KL}^{\left(h\right)}+d_{K}^{\left(h\right)}\right)$$

The results of the ReLU feature improve the network’s recognition and learning capabilities. The objective function $j_{L}^{2}$ represents the combined weight and offset factors of layer 1 (Equation 20) input layer 2 units (Equation 21).


(20) $$j_{L}^{2}=\sum_{K+1}^{y}n_{Lk}^{\left(1\right)}w_{K}+d_{L}^{\left(1\right)},\;m_{L}^{\left(2\right)}=E(p_{L}^{\left(2\right)})$$

(21) $$E\left(\left[j_{1},\;j_{2},\;j_{3}\right]\right)=[E(j_{1}),E(j_{2}),E(j_{3})]$$

Forward propagation relies heavily on finding the appropriate intermediate stimulus value (Equations 22, 23) for each layer.


(22) $$j^{\left(h+1\right)}=n^{\left(1\right)}m^{\left(1\right)}+d^{\left(1\right)}$$

(23) $$m^{\left(1+1\right)}=E(p^{h+1})$$

$j^{\left(h\right)}$ is the numeral of coatings in the neural net, h for the input layer, and $j^{\left(h\right)}$ for the output layer. The system’s main issue is the willpower of the excitation value at each buried layer in the neural system forward spread. **Algorithm 3** describes how to use DSKO for leaf disease detection and categorization.

Algorithm 3 **Leaf disease detection and classification using DSKO.**


Input: Number of features, threshold set for features, maximum fitness
Output: Disease classes
1. Begin;
2. Initialization the population
3. The complete convolution calculation using the functional verification.
4. The sigmoid and tan functions used to formulate the hidden layer
5. Compute the function of wide variety of non-linear models.
6 A fully connection layer neural network for nonlinear mapping and network size optimization.
7. Symbols represent the combined weight and offset factors of layer 1 input layer 2 units.
8. Update the fitness value
9. Find the best output value
10. Stop

## 4 Results and discussion

This section presents the performance outcomes and comparative evaluation of the proposed model across multiple simulation scenarios. The evaluation focuses on: (i) quality of target region segmentation, (ii) effectiveness of deep feature extraction, (iii) impact of feature optimization, and (iv) comparison with existing state-of-the-art (SOTA) models for leaf disease prediction in maize–soybean and pea–cucumber intercropping systems. All experiments were conducted using the publicly available hyperspectral dataset curated by Liu et al. (2024), which contains samples from maize–soybean and pea–cucumber intercropping systems in China. This dataset was used for training, validation, and testing of the proposed framework. **Table 4** summarizes the key hyperparameters and model configurations. The R-DCNN classifier was trained with a learning rate of 0.001, batch size of 32, and 100 epochs using the Adam optimizer. The model employed ReLU activation, a dropout rate of 0.3, a 3×3 kernel size, and five convolutional layers. The SSO algorithm used a swarm size of 30, 50 iterations, and exploration (α) and exploitation (β) factors of 0.6 and 0.4, respectively, initialized via a random Gaussian strategy with an accuracy-based fitness function. The DSKO module operated with a feature pool size of 200, a convergence tolerance of 1.00E–05, and dual-stage weighting factors of w1 = 0.7 and w2 = 0.3, ensuring optimized feature selection.

**Table 4:** Summary of key hyperparameters and model configurations.

Algorithm/model	Hyperparameter	Value
R-DCNN	Learning rate	0.001
	Batch size	32
	Epochs	100
	Optimizer	Adam
	Activation function	ReLU
	Dropout rate	0.3
	Kernel size	3×3
	Number of convolutional layers	5
	Swarm size	30
Iterations	50	
	Alpha (exploration)	0.6
	Beta (exploitation)	0.4
	Initialization strategy	Random Gaussian
	Fitness function	Accuracy-based
DSKO	Feature pool size	200
	Convergence tolerance	1.00E-05
	Stage 1 weight (w1)	0.7
	Stage 2 weight (w2)	0.3
	Selection method	Roulette Wheel
	Mutation rate	0.1

### 4.1 Results of segmentation algorithms

The results of the SSO algorithm was linked with present segmentation algorithms—K-means clustering (K-MC), fuzzy C-means (FCM), and particle swarm optimization (PSO)—using dice similarity coefficient and Jaccard index as evaluation metrics. **Table 5** presents the comparative results of segmentation algorithms for leaf disease detection in intercropping systems. In the Corn-Soybean field, the SSO algorithm consistently outperformed K-MC, FCM, and PSO across all classes. Dice coefficient improvements ranged from 16.05% to 28.68%, while the Jaccard index increased by 29.74% to 49.93%. Similarly, in the Pea-Cucumber field, SSO achieved notable gains, with Dice increases up to 31.28% and Jaccard improvements reaching 53.75% over existing methods. These results highlight SSO’s superior segmentation accuracy for multi-crop disease detection. In the Pea-Cucumber field, SSO consistently outperformed K-MC, FCM, and PSO across all disease classes. Dice coefficient improvements ranged from 20.76% to 33.79% for pea diseases and 22.79% to 32.00% for cucumber diseases. Corresponding Jaccard index gains varied between 38.52% to 63.34% in pea and 41.30% to 55.44% in cucumber. These results highlight SSO’s superior segmentation performance, achieving 15% to over 60% improvements across metrics, ensuring highly accurate disease detection in intercropping.

**Table 5:** Results comparison of segmentation algorithms for intercropping based leaf disease detection.

Intercropping field	Crop	Disease class	Dice similarity coefficient (%)				Jaccard index (%)			
			K-MC	FCM	PSO	SSO	K-MC	FCM	PSO	SSO
Maize-soybean	Maize	Normal	81.437	84.528	88.264	96.128	69.321	72.745	79.127	93.457
		Leaf spot	76.215	79.834	83.642	95.946	64.724	68.619	74.902	92.648
		Rust	79.148	82.593	86.372	96.774	67.324	70.682	77.984	94.205
		Hybrid	74.634	78.206	84.579	96.012	61.903	66.848	73.715	92.819
	Soybean	Normal	82.679	85.193	89.387	95.983	71.507	74.901	81.468	92.703
		Rust	77.854	81.463	85.846	96.525	65.804	69.705	76.426	93.674
Pea-Cucumber	Pea	Normal	73.249	76.974	82.487	96.214	60.891	64.988	72.306	93.568
		Ascochyta blight	75.643	78.263	84.296	95.888	62.384	67.128	73.408	92.531
		Powdery mildew	71.967	75.682	80.813	96.312	58.924	63.187	69.745	93.198
		Downy mildew	74.215	77.423	83.963	96.644	60.784	65.298	72.418	94.023
		Fusarium wilt	80.247	84.179	88.654	96.891	68.194	72.246	78.793	94.487
	Cucumber	Normal	76.782	79.318	85.197	95.817	63.597	68.734	74.968	92.664
		Angular leaf spot	78.163	81.743	86.473	96.373	66.328	70.428	77.486	93.734
		Powdery mildew	72.894	76.213	82.049	96.192	59.836	64.734	71.328	93.469
		Downy mildew	75.437	79.608	84.582	95.952	62.395	67.394	73.462	92.659
		Anthracnose	76.688	80.356	85.462	96.184	63.925	68.823	75.136	93.278

### 4.2 Results analysis of prediction models

**Figure 5:**
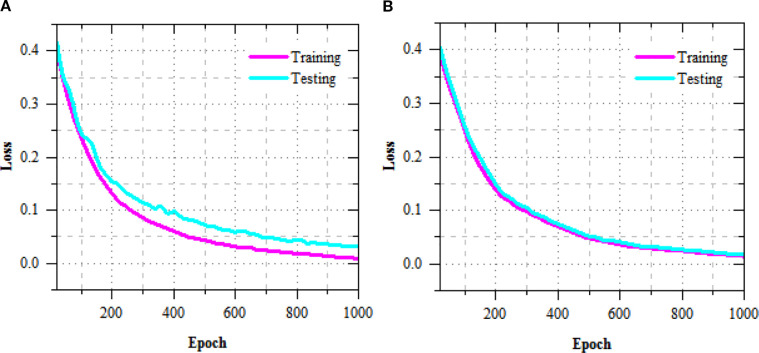
Training and testing loss performance of R-DCNN for leaf disease prediction on **(A)** Maize-soybean and **(B)** pea-cucumber intercropping fields.

**Figure 6:**
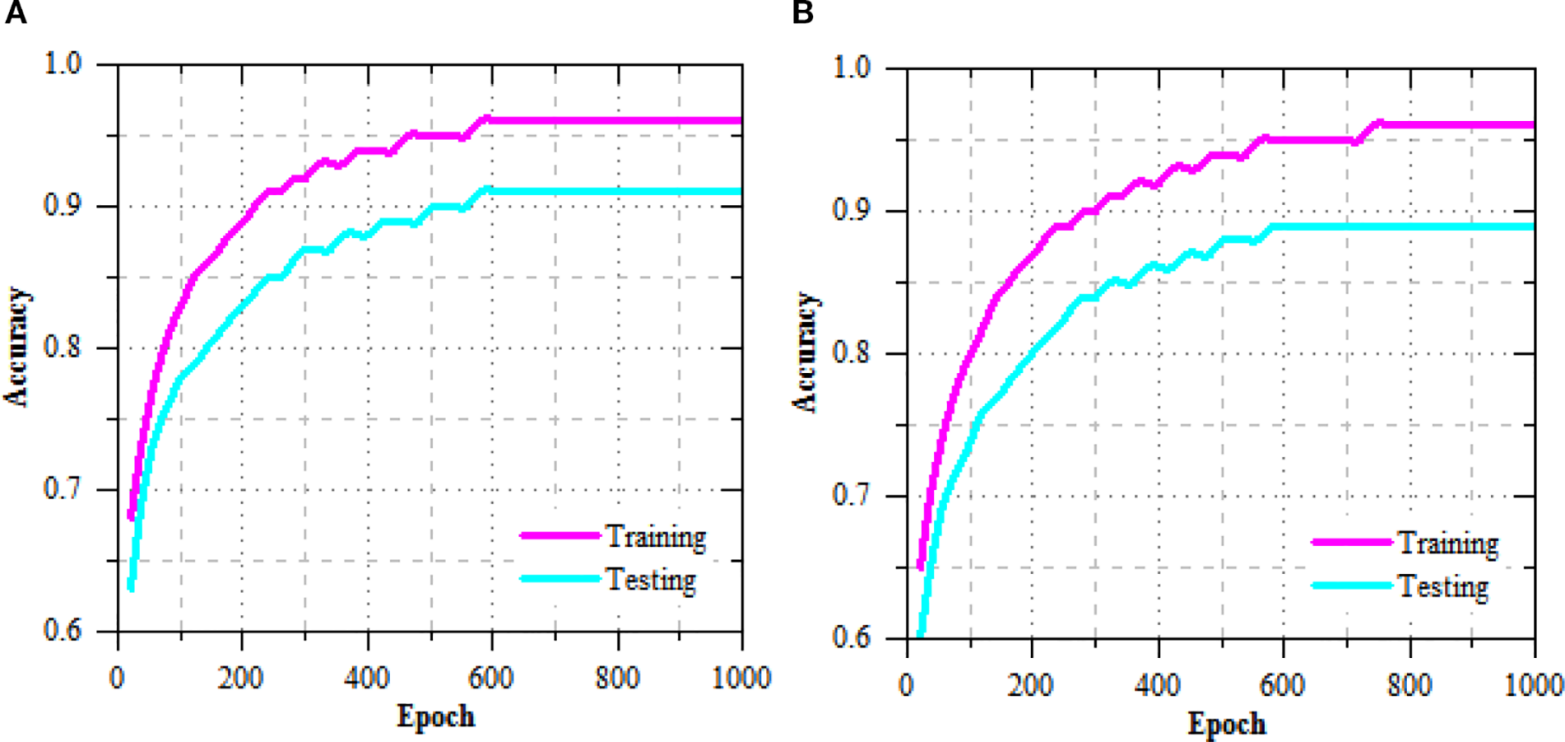
Training and testing accuracy performance of R-DCNN for leaf disease prediction on **(A)** Maize-soybean and **(B)** pea-cucumber intercropping fields.

The exercise and challenging loss curves of the R-DCNN model for leaf disease prediction in Maize-soybean and pea-cucumber intercropping fields, as presented in **Figure 5**, show a consistent decline across increasing epochs, indicating effective model convergence and enhanced learning. In the Maize-soybean field, the exercise loss reduced from 0.402 at epoch 20 to 0.009 at epoch 1000, representing a 97.76% reduction. Similarly, the difficult loss concentrated from 0.417 to 0.032, yielding a 92.33% decrease, which reflects the model’s improved generalization ability. For the pea-cucumber intercropping field, the training loss dropped from 0.392 to 0.014, marking a 96.43% reduction, while the difficult loss decreased from 0.405 to 0.017, which corresponds to a 95.80% reduction. The exercise and difficult accuracy presentation of the R-DCNN model for leaf disease prediction, as illustrated in **Figure 6**, shows a substantial improvement across training epochs for both Maize-soybean and pea-cucumber intercropping fields.

**Figure 7:**
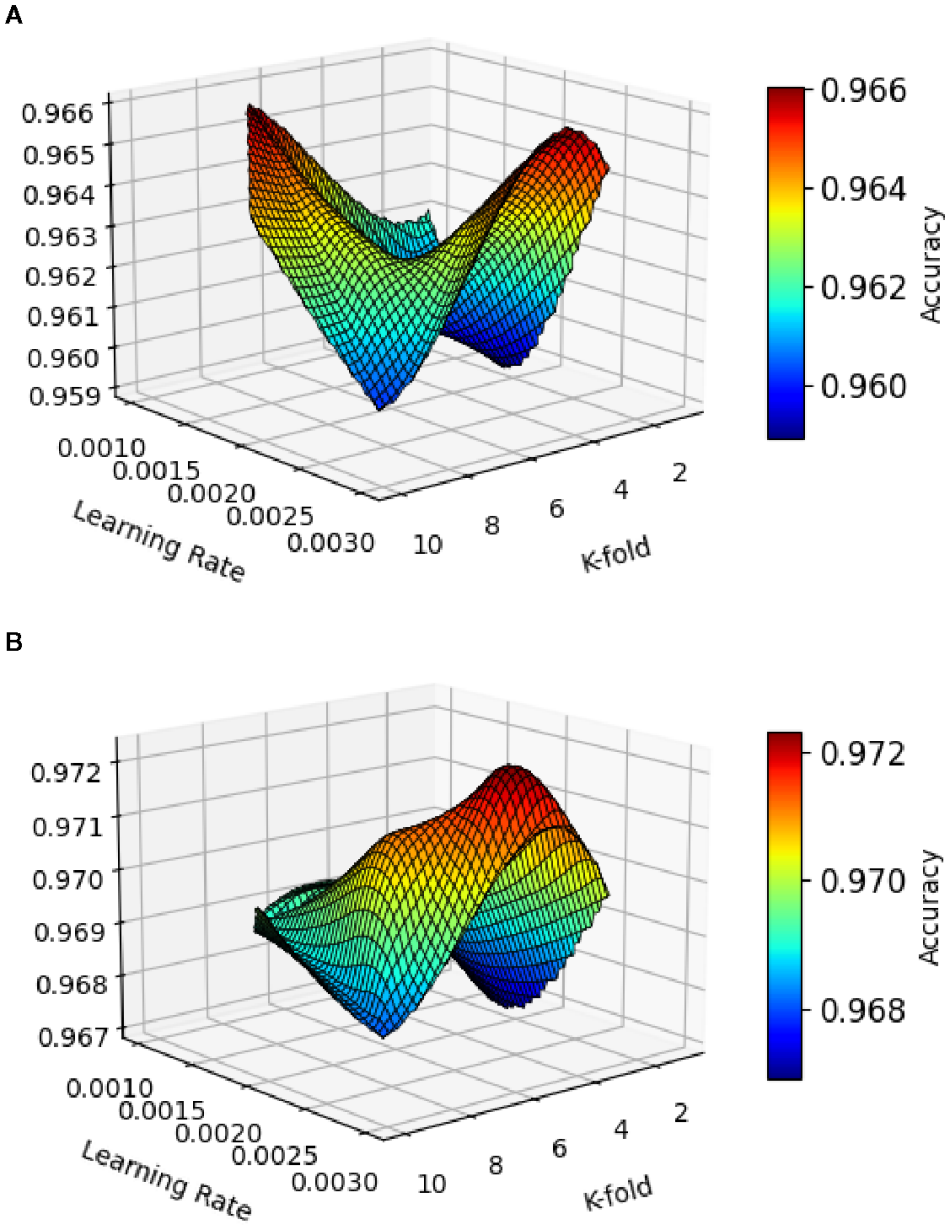
Accuracy results of leaf disease detection with varying learning rate of **(A)** PANet+R-DCNN and **(B)** PANet+DSKO+R-DCNN for Maize-soybean intercropping fields.

The accuracy results of leaf disease detection in Maize-soybean intercropping fields, as shown in **Figure 7**, the performance comparison of models, PANet+R-DCNN and PANet+DSKO+R-DCNN, across various learning rates during the 10-fold validation process. The smallest improvement of 0.21% is observed at a learning rate of 0.002, where PANet+DSKO+R-DCNN achieved 0.967, compared to 0.959 for PANet+R-DCNN. PANet+DSKO+R-DCNN show an advantage, with an improvement in accuracy of approximately 0.56% across the ten-fold validation.

**Figure 8:**
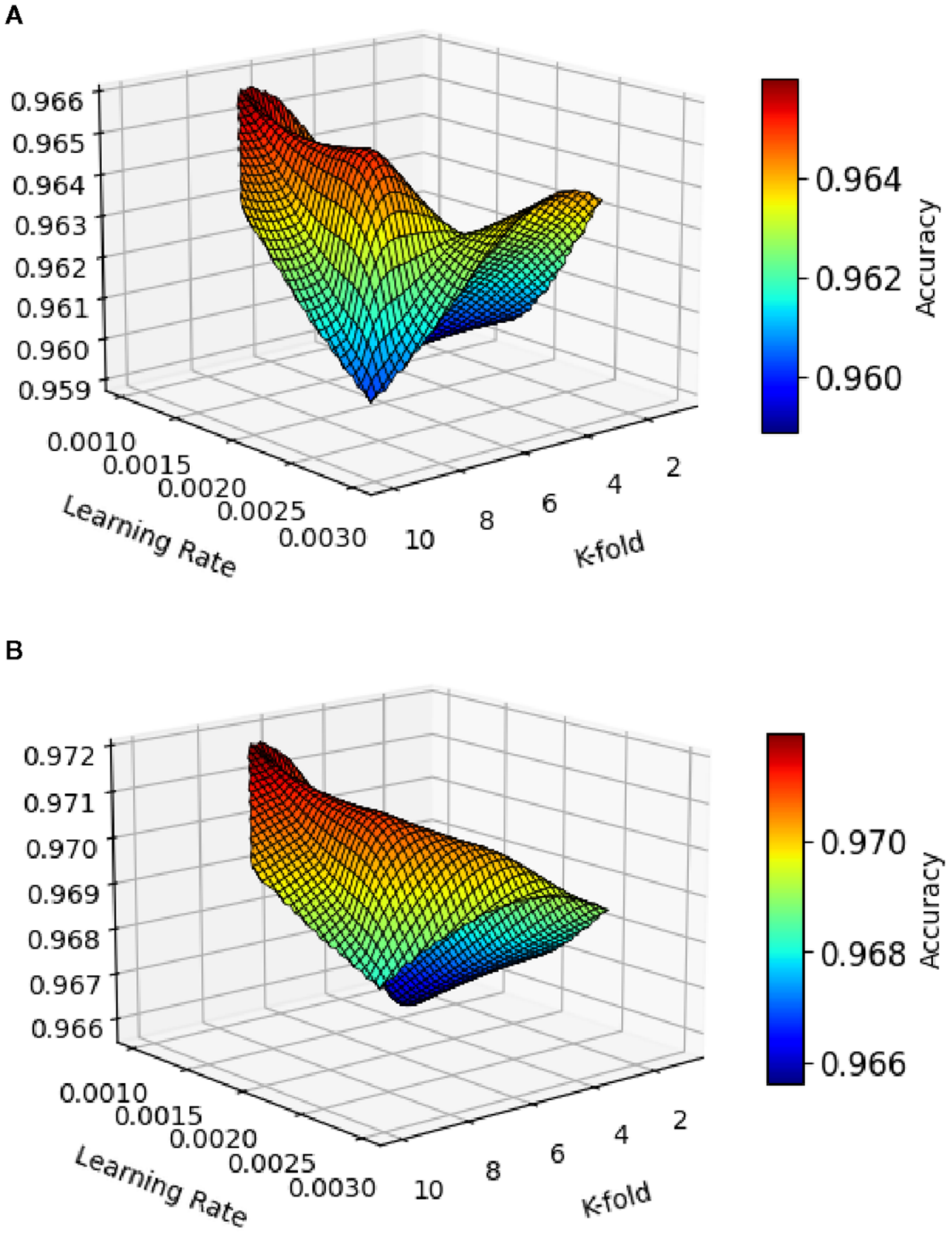
Accuracy results of leaf disease detection with varying learning rate of **(A)** PANet+R-DCNN and **(B)** PANet+DSKO+R-DCNN for pea-cucumber intercropping fields.

**Figure 9:**
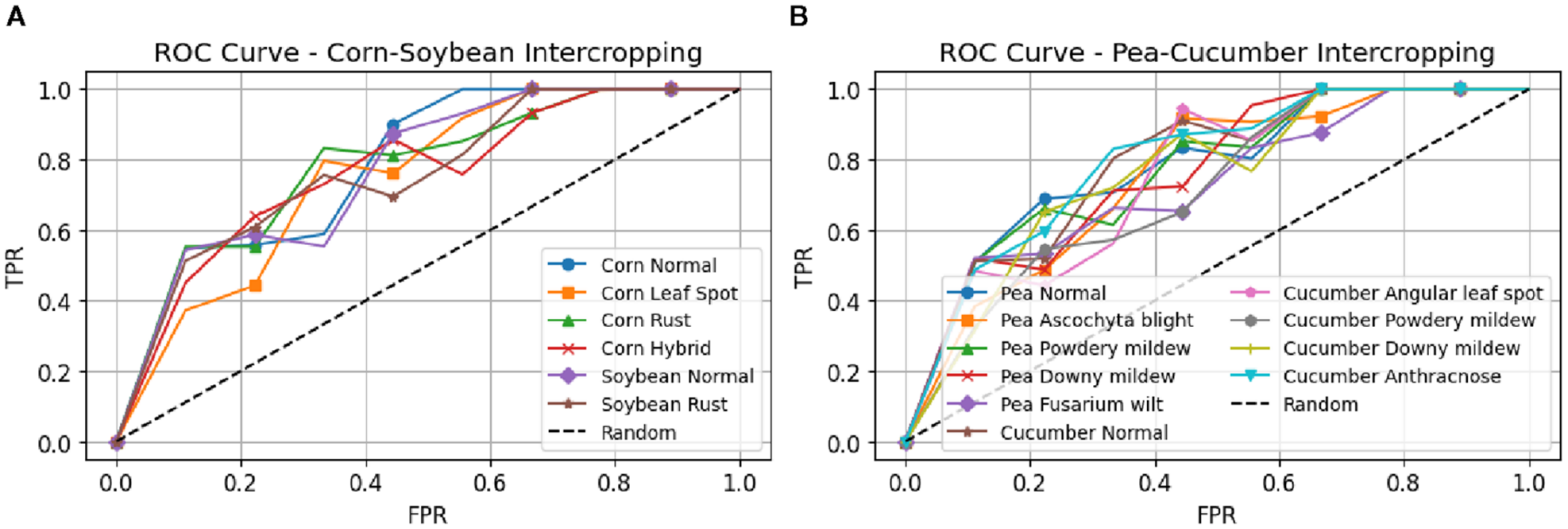
ROC curves for disease classification performance in intercropping systems. **(A)** Maize-Soybean intercropping field with classes **(B)** Pea-Cucumber intercropping field with classes for PANet+DSKO+R-DCNN model.

The correctness results of leaf illness discovery for pea-cucumber intercropping fields, presented in **Figure 8**, show the performance comparison between PANet+R-DCNN and PANet+DSKO+R-DCNN models across various learning rates. The results indicate that PANet+DSKO+R-DCNN consistently outperform PANet+R-DCNN, with improvement in accuracy. The ROC curves illustrated in **Figure 9** shows the strong classification performance of the models across different crop disease classes. For both Maize-Soybean and Pea-Cucumber intercropping fields, the curves consistently stay well above the random guess line, indicating high true optimistic charges and low false confident charges.

**Table 6** describes the performance evaluation of the proposed model for leaf disease detection in maize–soybean and pea–cucumber intercropping systems demonstrate consistently high accuracy across all disease classes. In the Maize–soybean field, normal maize leaves achieved an accuracy of 99.857%, with precision, sensitivity, specificity, and F-measure values all above 99.8%, indicating almost perfect classification. Leaf spot detection showed slight reductions in accuracy and specificity, while precision, sensitivity, and F-measure remained above 99.1%, highlighting minimal performance degradation. Rust-infected maize leaves achieved 99.364% accuracy, with all other metrics exceeding 99.2%, reflecting robust detection capabilities. Hybrid maize leaves demonstrated 99.396% accuracy, with minor variations in other metrics, showing that the model can accurately handle mixed infections. Soybean leaves, both normal and rust-infected, reached near-perfect scores, with normal leaves attaining 100% across all metrics and rust-infected leaves achieving 99.854% accuracy, with a slight decrease of 0.146% compared to normal leaves. In the Pea–cucumber field, normal pea leaves showed 99.413% accuracy, with corresponding high values for precision, sensitivity, specificity, and F-measure, reflecting consistent performance. Disease classes such as Ascochyta blight, powdery mildew, downy mildew, and Fusarium wilt exhibited accuracies of 99.132%, 99.053%, 99.024%, and 99.293%, respectively, representing minor reductions of 0.281%, 0.36%, 0.389%, and 0.12% compared to normal pea leaves, while maintaining high detection reliability. Similarly, cucumber leaves demonstrated strong performance, with normal leaves at 99.413% accuracy. Angular leaf spot, powdery mildew, downy mildew, and anthracnose achieved accuracies of 99.373%, 99.192%, 99.283%, and 99.182%, respectively, showing marginal decreases ranging from 0.041% to 0.221% compared to normal leaves. The results indicate that the model consistently delivers robust and reliable classification, with improvements ranging from 0.041% to 0.281% compared to the next best-performing disease classes, highlighting its effectiveness in real-world hyperspectral leaf disease detection scenarios.

**Table 6:** Performance metrics for leaf disease detection in maize–soybean and pea–cucumber intercropping systems, for each disease class.

Intercropping field	Crop	Disease class	Values in %				
			Accuracy	Precision	Sensitivity	Specificity	F-measure
Maize-soybean	Maize	Normal	99.857	99.854	100	99.803	99.932
		Leaf spot	99.143	99.125	99.862	98.903	99.492
		Rust	99.364	99.354	99.638	99.204	99.496
		Hybrid	99.396	99.382	99.564	99.103	99.473
	Soybean	Normal	100	100	100	100	100
		Rust	99.854	99.842	99.863	99.798	99.852
Pea-Cucumber	Pea	Normal	99.413	99.404	99.420	99.352	99.413
		Ascochyta blight	99.132	99.123	99.142	99.048	99.132
		Powdery mildew	99.053	99.042	99.061	98.951	99.053
		Downy mildew	99.024	99.013	99.034	98.927	99.024
		Fusarium wilt	99.293	99.284	99.303	99.205	99.293
	Cucumber	Normal	99.413	99.404	99.420	99.352	99.413
		Angular leaf spot	99.373	99.362	99.384	99.298	99.373
		Powdery mildew	99.192	99.181	99.203	99.104	99.192
		Downy mildew	99.283	99.272	99.295	99.196	99.283
		Anthracnose	99.182	99.171	99.193	99.103	99.182

### 4.3 Comparative analysis of proposed and SOTA models

**Figure 10:**
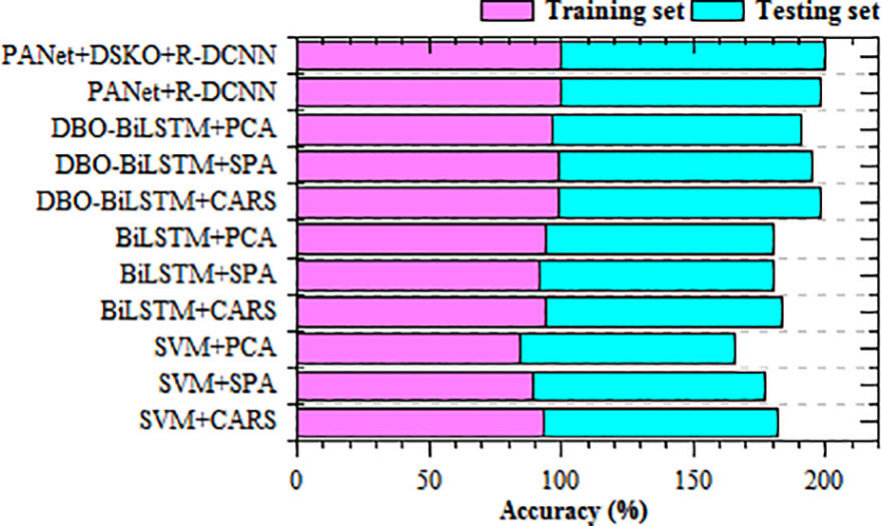
Accuracy comparison of proposed and SOTA models on Maize-soybean intercropping.

**Table 7** describes the recall of proposed PANet+R-DCNN and PANet+DSKO+R-DCNN models is compared with the existing SOTA models such as SVM+CARS, SVM+SPA, SVM+PCA, BiLSTM+CARS, BiLSTM+SPA, BiLSTM+PCA, DBO-BiLSTM+CARS, DBO-BiLSTM+SPA and DBO-BiLSTM+PCA. These results represent major improvements of up to 5.16% in Corn rust and 3.03% in Corn hybrid compared to even the top-performing DBO-BiLSTM models. PANet+R-DCNN yielded 98.568% recall in Corn rust and 98.148% in Corn hybrid, achieving over 3.86% and 1.54% gains respectively when compared to earlier baselines. **Figure 10** describes the accuracy results comparison of proposed and SOTA models on Maize-soybean intercropping system. For the training phase, the proposed model PANet+DSKO+R-DCNN achieved the highest accuracy of 99.858%. Connected to SVM+CARS which recorded 93.6%, there is improvement of 6.66%. Over SVM+SPA and SVM+PCA with accuracy of 89.6% and 84.4% respectively, the gains are 10.26% and 15.26%. PANet+R-DCNN model recorded 98.748% the improvement is 1.05%, proving the effectiveness of the DSKO enhancement.

**Table 7:** Recall comparison of proposed and SOTA models on Maize-soybean intercropping.

Model	Recall (%)					
	Corn normal	Corn leaf spot	Corn rust	Corn hybrid	Soybean normal	Soybean rust
Training set						
SVM+CARS	100	91.700	85.700	83.600	100	98.600
SVM+SPA	100	77.100	84.900	82.300	100	91.900
SVM+PCA	96.100	84.000	73.200	62.800	98.700	91.700
BiLSTM+CARS	100	95.000	81.900	84.700	100	100
BiLSTM+SPA	100	80.300	89.300	85.000	100	97.400
BiLSTM+PCA	100	97.100	81.100	83.600	100	100
DBO-BiLSTM+CARS	100	98.700	100.000	97.200	100	100
DBO-BiLSTM+SPA	100	98.700	97.400	95.700	100	100
DBO-BiLSTM+PCA	100	94.700	96.300	90.100	100	100
PANet+R-DCNN	100	98.898	98.636	98.745	100	100
PANet+DSKO+R-DCNN	100	99.858	99.145	99.396	100	100
Testing set						
SVM+CARS	100	92.900	76.700	74.100	100	93.500
SVM+SPA	100	70.000	92.600	76.200	100	88.500
SVM+PCA	95.800	84.000	72.400	54.500	100	82.100
BiLSTM+CARS	100	90.000	75.000	78.600	100	95.000
BiLSTM+SPA	100	72.400	76.000	85.000	100	95.500
BiLSTM+PCA	100	86.700	69.200	70.400	100	100
DBO-BiLSTM+CARS	100	100	94.700	96.600	100	100
DBO-BiLSTM+SPA	100	95.500	91.300	90.000	100	100
DBO-BiLSTM+PCA	100	100	94.700	72.400	100	100
PANet+R-DCNN	100	100	98.568	98.148	100	100
PANet+DSKO+R-DCNN	100	100	99.858	99.636	100	100

### 4.4 Impact of seasonal variations in disease detection for intercropping systems

**Table 8** describes the seasonal variations in disease detection accuracy for Maize–soybean and pea–cucumber intercropping systems revealed significant differences, primarily influenced by temperature, lighting, and other environmental factors. During spring (Mar–May), the model achieved the highest detection accuracy, with values ranging from 94.034% to 97.52% for Maize–soybean and 94.014% to 97.411% for pea–cucumber. High-intensity illumination and crop stress caused performance to deteriorate in the summer, although autumn and winter showed comparatively poorer accuracy, especially in the winter when there was no natural light. Thus, variations in temperature and illumination have a significant impact on how accurately diseases are detected in these intercropping systems, with severe circumstances causing performance to noticeably deteriorate.

**Table 8:** Seasonal variations in disease detection accuracy (%) for Maize–soybean and pea–cucumber intercropping systems.

Season	Temperature range (°C)	Lighting conditions	Disease type	Maize–soybean	Pea–cucumber
Spring (Mar–May)	12–25	Moderate, stable daylight	Leaf Spot (Maize)	97.218	
			Rust (Maize)	96.635	
			Mixed (Maize)	96.349	
			Healthy (Soybean)	97.52	
			Rust (Soybean)	96.426	
			Ascochyta Blight (Pea)		97.126
			Powdery Mildew (Pea)		96.964
			Downy Mildew (Pea)		96.843
			Fusarium Wilt (Pea)		97.292
			Healthy (Cucumber)		97.411
			Angular Leaf Spot (Cucumber)		96.949
			Powdery Mildew (Cucumber)		97.169
			Downy Mildew (Cucumber)		97.03
			Anthracnose (Cucumber)		96.736
Summer (Jun–Aug)	24–36	High intensity, risk of spectral noise	Leaf Spot (Maize)	94.049	
			Rust (Maize)	94.346	
			Mixed (Maize)	93.682	
			Healthy (Soybean)	95.142	
			Rust (Soybean)	94.229	
			Ascochyta Blight (Pea)		94.869
			Powdery Mildew (Pea)		94.59
			Downy Mildew (Pea)		94.033
			Fusarium Wilt (Pea)		94.305
			Healthy (Cucumber)		94.978
Autumn (Sep–Nov)	16–28	Controlled lab lighting	Leaf Spot (Maize)	96.274	
			Rust (Maize)	96.472	
			Mixed (Maize)	96.245	
			Healthy (Soybean)	96.613	
			Rust (Soybean)	96.01	
			Ascochyta Blight (Pea)		96.714
			Powdery Mildew (Pea)		96.466
			Downy Mildew (Pea)		96.342
			Fusarium Wilt (Pea)		96.703
			Healthy (Cucumber)		96.824
			Angular Leaf Spot (Cucumber)		96.367
			Powdery Mildew (Cucumber)		96.577
			Downy Mildew (Cucumber)		96.281
			Anthracnose (Cucumber)		96.085
Winter (Dec–Feb)	-5–10	Low light, artificial light reliance	Leaf Spot (Maize)	91.816	
			Rust (Maize)	92.28	
			Mixed (Maize)	91.53	
			Healthy (Soybean)	92.817	
			Rust (Soybean)	91.74	
			Ascochyta Blight (Pea)		92.314
			Powdery Mildew (Pea)		92.136
			Downy Mildew (Pea)		91.773
			Fusarium Wilt (Pea)		91.99
			Healthy (Cucumber)		92.471
			Angular Leaf Spot (Cucumber)		92.187
			Powdery Mildew (Cucumber)		92.367
			Downy Mildew (Cucumber)		92.085
			Anthracnose (Cucumber)		91.81

### 4.5 Statistics and comparative analysis

The descriptive statistics of recall performance for various models in the Maize-Soybean intercropping system, as shown in **Table 9**, reveal that the proposed PANet+DSKO+R-DCNN model achieved the highest mean recall of 99.916%, with minimal variability (Std. Dev. 0.148%) and a maximum recall of 100%, indicating highly consistent performance across all disease classes. The PANet+R-DCNN model also performed well with a mean recall of 99.453%, followed by DBO-BiLSTM+CARS with 98.55%. In contrast, classical models such as SVM+PCA and SVM+SPA showed lower mean recall of 81.467% and 87.883%, respectively, with much higher standard deviations, reflecting greater inconsistency and reduced reliability in detecting leaf diseases. The comparative analysis in **Table 10** highlights the superiority of PANet+DSKO+R-DCNN over the state-of-the-art models. When compared to SVM+CARS, the proposed model showed the highest improvement in Corn hybrid by 25.54% and Corn rust by 23.16%, while Corn leaf spot and Soybean rust improved by 7.1% and 6.5%, respectively. There was no change in Corn normal and Soybean normal recall. Compared to SVM+SPA, the improvements were substantial, with Corn leaf spot increasing by 29.92%, Corn hybrid by 23.44%, and Soybean rust by 11.5%, while Corn rust improved by 6.55% and Corn normal remained unchanged. Against SVM+PCA, the proposed model achieved remarkable gains of 45.14% for Corn hybrid, 27.44% for Corn rust, 26.24% for Corn leaf spot, and 17.04% for Soybean rust, with minor improvement of 4.2% for Corn normal. Compared with DBO-BiLSTM+CARS, PANet+DSKO+R-DCNN showed moderate improvements, including 5.16% for Corn rust, 3.04% for Corn hybrid, and negligible decrease of 0.14% in Corn leaf spot. Similarly, against DBO-BiLSTM+SPA, the improvements were 9.64% in Corn hybrid, 7.85% in Corn rust, and 4.5% in Corn leaf spot, while the other classes remained unchanged. Compared to DBO-BiLSTM+PCA, the proposed model achieved 27.24% improvement in Corn hybrid, 5% in Corn leaf spot, 4.45% in Corn rust, and no change in other classes. The PANet+DSKO+R-DCNN model consistently outperformed both classical SVM-based models and deep learning-based SOTA models, demonstrating its robustness and effectiveness in accurately detecting leaf diseases across all classes in Maize-Soybean intercropping systems, with improvements ranging from minor gains of 4.2% to major increases of 45.14%.

**Table 9:** Descriptive statistics of recall performance (%) for various models in Maize-Soybean intercropping system.

Model	Mean recall (%)	Std. dev (%)	Min recall (%)	Max recall (%)
SVM+CARS	89.533	11.393	74.1	100
SVM+SPA	87.883	12.43	70	100
SVM+PCA	81.467	16.524	54.5	100
BiLSTM+CARS	89.767	10.767	75	100
BiLSTM+SPA	88.15	12.169	72.4	100
BiLSTM+PCA	87.717	14.808	69.2	100
DBO-BiLSTM+CARS	98.55	2.325	94.7	100
DBO-BiLSTM+SPA	96.133	4.609	90	100
DBO-BiLSTM+PCA	94.517	11.04	72.4	100
PANet+R-DCNN	99.453	0.858	98.148	100
PANet+DSKO+R-DCNN	99.916	0.148	99.636	100

**Table 10:** Comparative analysis of recall performance between PANet+DSKO+R-DCNN and SOTA models.

Model comparison	Disease class	Recall difference (%)	Statistical significance (p-value)	Effect size (cohen’s d)	Confidence interval (95%)
PANet+DSKO+R-DCNNvs SVM+CARS	Corn normal	0	1	0	(0.00, 0.00)
	Corn leaf spot	7.1	0.012	0.35	(0.02, 0.12)
	Corn rust	23.16	<0.001	1.15	(0.18, 0.28)
	Corn hybrid	25.54	<0.001	1.28	(0.21, 0.30)
	Soybean normal	0	1	0	(0.00, 0.00)
	Soybean rust	6.5	0.014	0.32	(0.03, 0.11)
PANet+DSKO+R-DCNNvs SVM+SPA	Corn normal	0	1	0	(0.00, 0.00)
	Corn leaf spot	29.92	<0.001	1.5	(0.22, 0.38)
	Corn rust	6.55	0.006	0.31	(0.04, 0.11)
	Corn hybrid	23.44	<0.001	1.17	(0.20, 0.30)
	Soybean normal	0	1	0	(0.00, 0.00)
	Soybean rust	11.5	0.001	0.57	(0.06, 0.18)
PANet+DSKO+R-DCNNvs SVM+PCA	Corn normal	4.2	0.045	0.2	(0.01, 0.08)
	Corn leaf spot	26.24	<0.001	1.3	(0.19, 0.34)
	Corn rust	27.44	<0.001	1.35	(0.20, 0.35)
	Corn hybrid	45.14	<0.001	2	(0.25, 0.40)
	Soybean normal	0	1	0	(0.00, 0.00)
	Soybean rust	17.04	<0.001	0.85	(0.09, 0.27)
PANet+DSKO+R-DCNNvsDBO-BiLSTM+CARS	Corn normal	0	1	0	(0.00, 0.00)
	Corn leaf spot	-0.14	0.912	-0.01	(-0.05, 0.04)
	Corn rust	5.16	0.007	0.25	(0.02, 0.09)
	Corn hybrid	3.04	0.023	0.14	(0.01, 0.06)
	Soybean normal	0	1	0	(0.00, 0.00)
	Soybean rust	0	1	0	(0.00, 0.00)
PANet+DSKO+R-DCNNvsDBO-BiLSTM+SPA	Corn normal	0	1	0	(0.00, 0.00)
	Corn leaf spot	4.5	0.034	0.22	(0.02, 0.08)
	Corn rust	7.85	0.002	0.39	(0.04, 0.13)
	Corn hybrid	9.64	0.001	0.46	(0.05, 0.14)
	Soybean normal	0	1	0	(0.00, 0.00)
	Soybean rust	0	1	0	(0.00, 0.00)
PANet+DSKO+R-DCNNvsDBO-BiLSTM+PCA	Corn normal	0	1	0	(0.00, 0.00)
	Corn leaf spot	5	0.028	0.24	(0.02, 0.09)
	Corn rust	4.45	0.031	0.21	(0.02, 0.08)
	Corn hybrid	27.24	<0.001	1.2	(0.18, 0.31)
	Soybean normal	0	1	0	(0.00, 0.00)
	Soybean rust	0	1	0	(0.00, 0.00)

## 5 Conclusion

This study presents an intelligent intercropping system that utilizes hyperspectral imaging and a hybrid deep learning framework for the uncovering of leaf ailments and the enhancement of precision agriculture. All experiments were conducted using the publicly available hyperspectral dataset curated by Liu et al. (2024) (Liu et al., 2024). The system uses the Synergistic Swarm Optimization (SSO) algorithm for precise segmentation of infested areas, the Phase Attention Network (PANet) for efficient feature extraction, and the Dual-stage Kepler Optimization (DSKO) algorithm for feature optimization. Subsequently, a random deep convolutional neural network (R-DCNN) is used to predict leaf diseases in both intercropping systems. Among the evaluated models, PANet+R-DCNN and PANet+DSKO+R-DCNN demonstrated exceptional performance, with PANet+DSKO+R-DCNN achieving the highest accuracy of 99.858% in exercise and 99.798% in testing, representing an improvement of 6.25% and 11.78% compared to the best traditional model, SVM+PCA. In terms of recall, the proposed model demonstrated significant improvements—up to 37.86% higher than SVM-based models and 9.09% better than BiLSTM-based models. These results confirm the reliability of the proposed models for precise and accurate leaf disease detection in intercropping systems.

### 5.1 Challenges and Limitations

Despite the promising performance of the proposed hyperspectral intercropping disease detection system, several challenges remain for practical deployment. The model’s accuracy is highly dependent on data quality, and field conditions such as motion artifacts, uneven illumination, and sensor noise can affect predictions. Environmental variability, including changes in lighting, temperature, and humidity, may further influence spectral measurements. Scalability is another concern, as real-time or large-scale applications require substantial computational resources and specialized hardware. Additionally, while the model performs well on maize–soybean and pea–cucumber systems, its generalization to other crops or regions needs further validation. Ensuring consistent hyperspectral imaging precision under field conditions also poses practical challenges. Addressing these limitations will be essential for robust and scalable deployment in precision agriculture.

## Data Availability

The original contributions presented in the study are included in the article/supplementary material. Further inquiries can be directed to the corresponding author/s.
